# Unfolding the Challenges To Prepare Single Crystalline Complex Oxide Membranes by Solution Processing

**DOI:** 10.1021/acsami.4c05013

**Published:** 2024-07-05

**Authors:** Pol Salles, Roger Guzman, Huan Tan, Martí Ramis, Ignasi Fina, Pamela Machado, Florencio Sánchez, Gabriele De Luca, Wu Zhou, Mariona Coll

**Affiliations:** † Institut de Ciència de Materials de Barcelona, ICMAB-CSIC, Campus UAB, 08193 Bellaterra, Spain; ‡ School of Physical Sciences, University of Chinese Academy of Sciences, Beijing 100049, China

**Keywords:** BiFeO_3_, Sr_3_Al_2_O_6_, sacrificial layer, chemical solution deposition, epitaxy, freestanding, oxide thin film

## Abstract

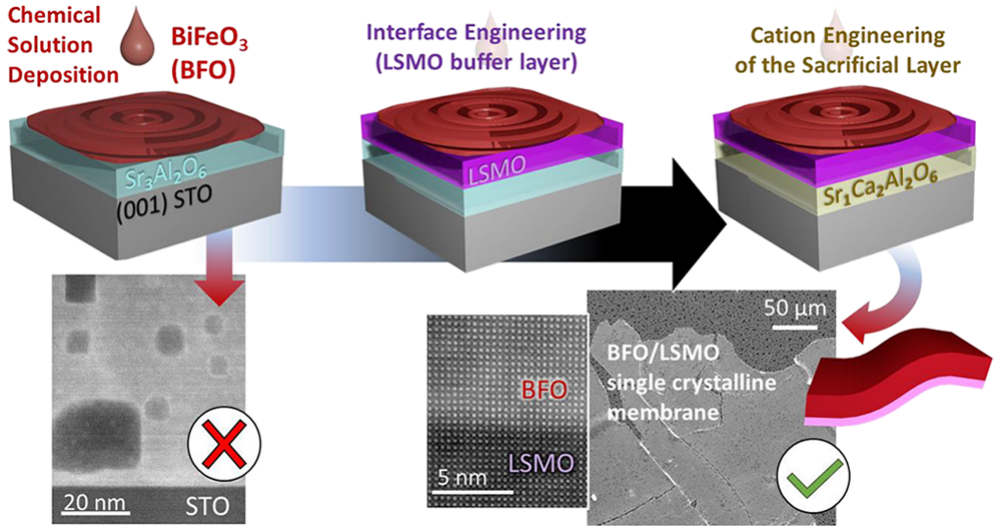

The ability to prepare single crystalline complex oxide freestanding membranes has opened a new playground to access new phases and functionalities not available when they are epitaxially bound to the substrates. The water-soluble Sr_3_Al_2_O_6_ (SAO) sacrificial layer approach has proven to be one of the most promising pathways to prepare a wide variety of single crystalline complex oxide membranes, typically by high vacuum deposition techniques. Here, we present solution processing, also named chemical solution deposition (CSD), as a cost-effective alternative deposition technique to prepare freestanding membranes identifying the main processing challenges and how to overcome them. In particular, we compare three different strategies based on interface and cation engineering to prepare CSD (00l)-oriented BiFeO_3_ (BFO) membranes. First, BFO is deposited directly on SAO but forms a nanocomposite of Sr–Al–O rich nanoparticles embedded in an epitaxial BFO matrix because the Sr–O bonds react with the solvents of the BFO precursor solution. Second, the incorporation of a pulsed laser deposited La_0.7_Sr_0.3_MnO_3_ (LSMO) buffer layer on SAO prior to the BFO deposition prevents the massive interface reaction and subsequent formation of a nanocomposite but migration of cations from the upper layers to SAO occurs, making the sacrificial layer insoluble in water and withholding the membrane release. Finally, in the third scenario, a combination of LSMO with a more robust sacrificial layer composition, SrCa_2_Al_2_O_6_ (SC_2_AO), offers an ideal building block to obtain (001)-oriented BFO/LSMO bilayer membranes with a high-quality interface that can be successfully transferred to both flexible and rigid host substrates. Ferroelectric fingerprints are identified in the BFO film prior and after membrane release. These results show the feasibility to use CSD as alternative deposition technique to prepare single crystalline complex oxide membranes widening the range of available phases and functionalities for next-generation electronic devices.

## Introduction

Epitaxial complex oxide thin films are gaining tremendous interest to boost or even outperform current electronic devices thanks to their extraordinary functional properties, including multiferroicity, ferroelectricity, colossal magnetoresistance, superconductivity, and metal–insulator transition.^1^ Among the family of complex oxides, BiFeO_3_ (BFO) is a multiferroic material that can also show photoinduced effects, being thus an attractive candidate to be used in next-generation electronic devices for applications in information storage, spintronics, sensors, actuators, and optoelectronics.^2−8^ Until recently, the preparation of epitaxial complex oxide films, such as BFO, was restricted to specific substrates that can stand high-temperature treatments, most of them on rigid surfaces or with a small bending strain.^9−11^ Therefore, the use of these substrates dramatically narrowed the potential applications of the epitaxial complex oxide films grown on top. The development of fabrication approaches that allow detaching the complex oxide from the growth substrate and freely handling it has opened a new ground of research.^12,13^ It provides an unparalleled opportunity to manipulate these structures by applying extreme deformations,^14^ unlocking the system’s elastic response upon applying an external stimuli,^15−17^ creating artificial architectures with emerging phenomena at the new interfaces,^18^ and integrating them with the platform of choice.^19−23^

Among the various existing approaches, the use of the water-soluble Sr_3_Al_2_O_6_ (SAO) sacrificial layer and derived compositions is quite extended to ultimately obtain crystalline and single-oriented complex oxide membranes.^19,24,25^ Successful preparation of (00l)-BFO freestanding membranes using a SAO sacrificial layer has been recently reported using high vacuum deposition techniques. Large flexural deformations with associated giant polarization and continuously controllable photoconductance in bended substrates are some pioneering examples of the uniqueness of BFO freestanding membranes.^26−28^ Note that the properties of the complex oxides, here BFO, are extremely sensitive to chemical and structural modifications, and the deposition technique also plays a role in it.^29−31^ Chemical solution deposition (CSD), also named solution processing, is an ambient-pressure and potentially scalable thin film growth process that offers large versatility to modify the chemical composition and stoichiometry of the material by properly selecting the chemical precursors and convert them to crystalline phases through a thermal treatment.^32^ The CSD films undergo a different growth mechanism than those prepared by vacuum techniques and can deliver distinct properties.^33^

It has been demonstrated that the SAO sacrificial layer can be successfully prepared by CSD^34^ but upon air exposure its surface becomes amorphous and defines the crystallinity of the oxide membrane.^35^ More recently, it has been found that the use of a cation-engineered CSD-Sr_3–*x*_Ca_*x*_Al_2_O_6_ (*x* ≤ 3) sacrificial layer provides higher ambient stability and improved surface crystallinity compared to pristine SAO while expanding the platform of available lattice parameters of sacrificial layers to obtain single-crystalline membranes.^36^ In this exciting framework, we aim to investigate the synthesis and structure of crystalline CSD-BFO membranes by exploring three different scenarios. First, we present an all-CSD landscape in which BFO films are deposited on SAO films. Second, we examine the use of pulsed laser deposited (PLD) La_0.7_Sr_0.3_MnO_3_ (LSMO) as a buffer layer on CSD-SAO to subsequently deposit CSD-BFO. Third, the combination of PLD-LSMO and CSD-SrCa_2_Al_2_O_6_ (SC_2_AO) is adopted to prepare epitaxial CSD-BFO films. Meticulous structural analysis by means of X-ray diffraction (XRD) and Scanning Transmission Electron Microscopy (STEM) combined with electron energy loss spectroscopy (EELS) is presented for the three different heterostructures and the corresponding (00l)-oriented membranes. A comprehensive study of the interface quality, film crystallinity, and lattice distortion demonstrates that the use of PLD-LSMO/CSD-SC_2_AO heterostructure is a suitable building block to deliver relaxed (00l)-oriented CSD-BFO membranes with ferroelectric behavior, putting forward a methodology that could be easily extended to other perovskite oxide compositions.

## Results and Discussion

### All-Solution Processed: CSD-BFO/CSD-SAO

**Figure 1:**
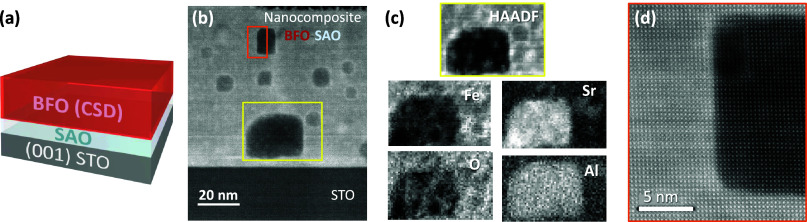
(a) BFO/SAO//STO heterostructure; (b) Z-contrast HAADF-STEM cross-sectional images of the CSD-BFO/CSD-SAO//STO; (c) EELS elemental mapping of Fe L-edge, Sr L-edge, O K-edge, and Al K-edge performed in the yellow-squared area from (b); (d) high-resolution image at the BFO-SAO nanocomposite interface.

75 nm CSD-BFO films were deposited on a heterostructure consisting of a 20 nm CSD-SAO sacrificial layer on a SrTiO_3_ (STO) single-crystal substrate (BFO/SAO//STO), Figure 1a. The small lattice mismatch between BFO and SAO (ϵ ∼ 0.2%) hindered the unambiguous identification of the formation of (00l) BFO and (00l)-oriented SAO by standard θ–2θ XRD, see Figure S1. Nonetheless, Z-contrast high-angle annular dark-field scanning transmission electron microscopy (HAADF-STEM) images identified the formation of a nanocomposite in which epitaxial and strongly faceted Sr–Al–O rich nanoparticles were embedded in an epitaxial BFO matrix instead of the expected discrete BFO and SAO layers, Figure 1b–d, which precluded the release of a freestanding membrane. Considering that PLD-BFO membranes can be readily achieved from PLD-SAO,^26^ it is suggested that here the solvent of the BFO precursor solution reacted with the Sr–O bonds from SAO, which are easy to hydrolyze,^37^ and triggered the reactivity of the whole system.

### Interface Engineering with a Buffer Layer: CSD-BFO/PLD-LSMO/CSD-SAO

**Figure 2:**
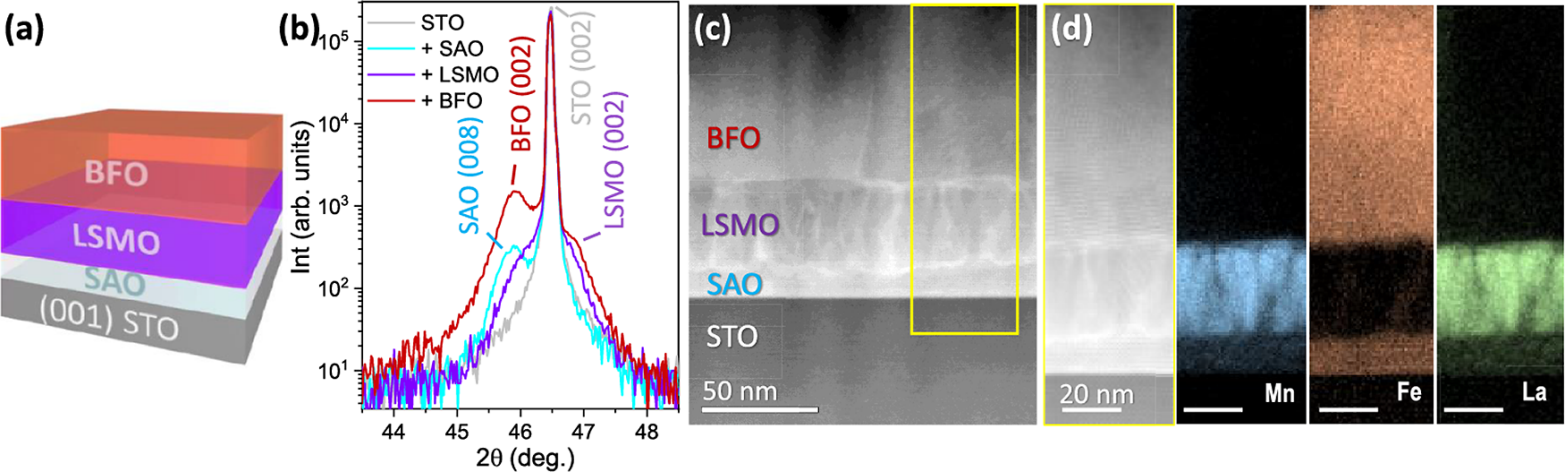
(a) BFO/LSMO/SAO//STO heterostructure; (b) XRD θ–2θ scan after the consecutive growth of SAO, LSMO, and BFO on a (001) STO substrate. The + sign in the legend indicates the sequential addition of the oxide component in the heterostructure BFO/LSMO/SAO//STO; (c) Z-contrast HAADF-STEM cross-section of the final heterostructure. The yellow rectangle corresponds to (d) EELS elemental mapping of Mn, Fe, and La along the heterostructure cross-section.

To prevent this massive interface reaction, the deposition of CSD-BFO was attempted on 60 nm PLD-LSMO-buffered SAO//STO,^36^Figure 2a. In this case, the (00l) Bragg reflections of BFO, LSMO, and SAO were identified next to the (002) STO reflection; see Figure 2b. In addition, it is noted that the (008) SAO reflection modifies in peak intensity and position after the sequential deposition of LSMO and BFO. This variation could be attributed to slight reactivity during deposition, and it is discussed below. From the Z-contrast HAADF-STEM image of this heterostructure, three discrete layers are clearly identified, Figure 2c, being a significant improvement from the nanocomposite scenario shown in Figure 1. EELS elemental mapping reveals a strong migration of Fe species and a few Mn and La to the SAO sacrificial layer; see Figure 2d. It is likely that the ion migration forms a complex phase consisting of SAO with Mn, Fe, and La, which can explain the changes previously identified in the (008) SAO reflection. As a result, the sacrificial layer becomes insoluble in water preventing the release of the BFO/LSMO bilayer upon water immersion.

### Cation Engineering of the Sacrificial Layer: CSD-BFO/PLD-LSMO/CSD-SC_2_AO

To build a more robust system against cation migration, a Ca^2+^-doped SAO (SrCa_2_Al_2_O_6_, SC_2_AO) sacrificial layer was prepared. This composition has been earlier demonstrated to be more stable than pristine SAO as Ca–O bonds are less prone to hydrolyze than Sr–O.^36^ Nonetheless, direct deposition of CSD-BFO on CSD-SC_2_AO//STO resulted in major reactivity, similar to the previously described scenario in BFO/SAO, see Figure S2. Consequently, it impeded the etching of the sacrificial layer.

**Figure 3:**
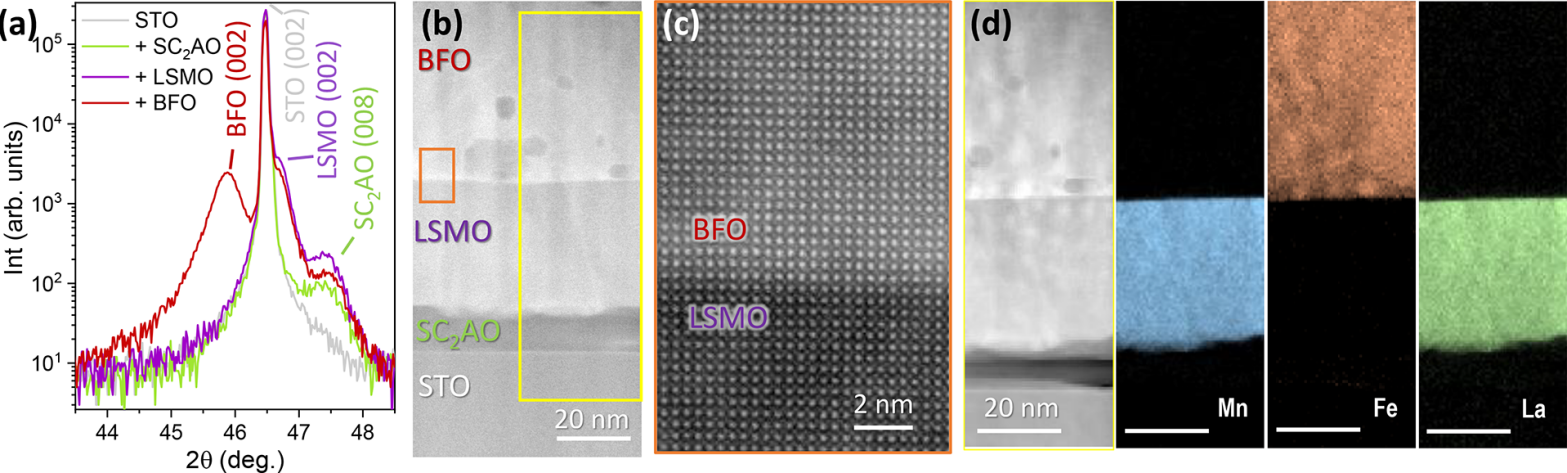
(a) XRD θ–2θ scans after the sequential growth of SC_2_AO (20 nm) on STO, followed by the deposition of LSMO (60 nm) on SC_2_AO//STO, and the BFO (75 nm) on LSMO/SC_2_AO//STO. The + sign in the legend indicates the last layer added to the heterostructure; (b) Z-contrast HAADF-STEM cross-section of the final BFO/LSMO/fSC_2_AO//STO heterostructure: (c) high-resolution magnification of the BFO/LSMO interface; (d) EELS elemental mapping of Mn, Fe, and La throughout the heterostructure cross-section.

Then, the BFO films were prepared on LSMO/SC_2_AO//STO. The XRD θ–2θ scans acquired after the deposition of each layer show the appearance of the (00l) Bragg reflections for BFO, LSMO, and SC_2_AO, Figure 3a, revealing a preferred *c*-axis growth of the multilayered system where the 2θ position of the (008) SC_2_AO reflection is maintained after LSMO deposition, as a sign of higher film stability. Further XRD structural analysis from this heterostructure identifies that the LSMO film is partially strained (+0.3%), whereas the BFO is fully relaxed with *a* = 3.96 Å, matching the bulk value.^38^ This is not surprising considering that CSD films with similar thickness tend to grow fully relaxed through the formation of structural defects.^30,39^ Cross-section STEM reveals the growth of three discrete layers in which BFO presents few Fe-rich secondary phases,^30,33,40^Figure 3b. Image magnification at the BFO/LSMO interface shows the atomic structure of both phases, corroborating their epitaxial relationship with an atomically sharp interface, Figure 3c. Unlike the previous scenarios, here EELS elemental mapping confirms the absence of both cation interdiffusion and Fe migration to the sacrificial layer; see Figure 3d. The inhomogeneous appearance of SC_2_AO in Figure 3d results from its structural softness when it is exposed to the electron beam.

Thus, the chemical formulation of this CSD-BFO solution requires both PLD-LSMO buffer and robust CSD-SC_2_AO sacrificial to avoid system reactivity and preserve the integrity of the heterostructure.

**Figure 4:**
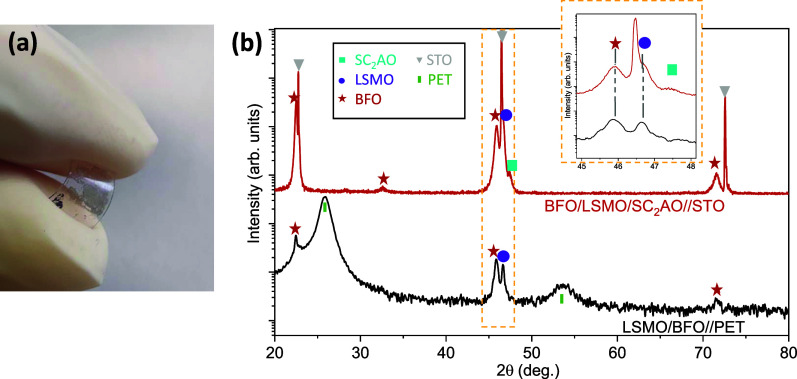
(a) Photography of a BFO(75 nm)/LSMO(60 nm) flake transferred on the PET substrate. (b) XRD θ–2θ scan of a BFO/LSMO/SC_2_AO//STO heterostructure and the released BFO/LSMO membrane on a PET support. The broad peaks at 26.2 and 53.7° correspond to the PET support. Inset corresponding to the 2θ range of the (002) STO Bragg reflection.

This heterostructure was further investigated to obtain single crystalline membranes. Upon attaching a polyethylene terephthalate (PET) sheet on the BFO/LSMO/SC_2_AO//STO system and immersing it in Milli-Q water, flexible BFO/LSMO membranes were obtained, see Figure 4a. XRD θ–2θ scans of the heterostructure and the bilayer membrane on PET are shown in Figure 4b. Both spectra show the (002) Bragg reflections for BFO and LSMO, confirming that the bilayer membranes retain the epitaxial relationship on flexible substrates. A minor contribution at 2θ = 32° from the (110) BFO Bragg reflection is observed in the heterostructure indicating that few BFO grains grow misoriented. The appearance of this trace is mostly attributed to the multideposition process to obtain BFO on PLD-LSMO/CSD-SC_2_AO//STO, which involves roughened CSD film surfaces compared with the bare STO substrate. In fact, CSD-BFO films grown on atomically flat STO substrates do not show this contribution.^30,41^ The 2θ positions for BFO and LSMO Bragg reflections in the flexible system do not change compared to the rigid substrate, see inset Figure 4b. Therefore, macroscopically, the BFO is fully relaxed, and the LSMO film is partially strained. Differently, note that when crystalline PLD-LSMO single membranes are prepared, they fully relax upon release through the formation of morphological defects such as cracks and wrinkles.^36^ The difference in the LSMO strain state from single to bilayer architecture can be assigned to the fact that the BFO layer pins partially strained the LSMO film, as previously identified in Ba_1–*x*_Sr_*x*_TiO_3_ membranes on SrRuO_3_ electrodes.^42^ The texture quality of the bilayer membrane has been assessed from the Δω (002)_BFO_ and Δω (002)_LSMO_ resulting in a full width at half-maximum (fwhm) of 1.30 ± 0.02° and 0.90 ± 0.02°, respectively. These values are similar to those obtained from the films in the heterostructure and slightly larger than those for the films grown on atomically flat single-crystal STO substrate, see Table S1. Considering that the surface roughness of the PLD-LSMO/CSD-SC_2_AO (2.1 ± 0.1 nm) and CSD-SC_2_AO (0.9 ± 0.2 nm) is remarkably higher than the atomically flat STO single crystals, it is very likely that the roughness of the underlying layer can contribute to an increase in the Δω of the films.

**Figure 5:**
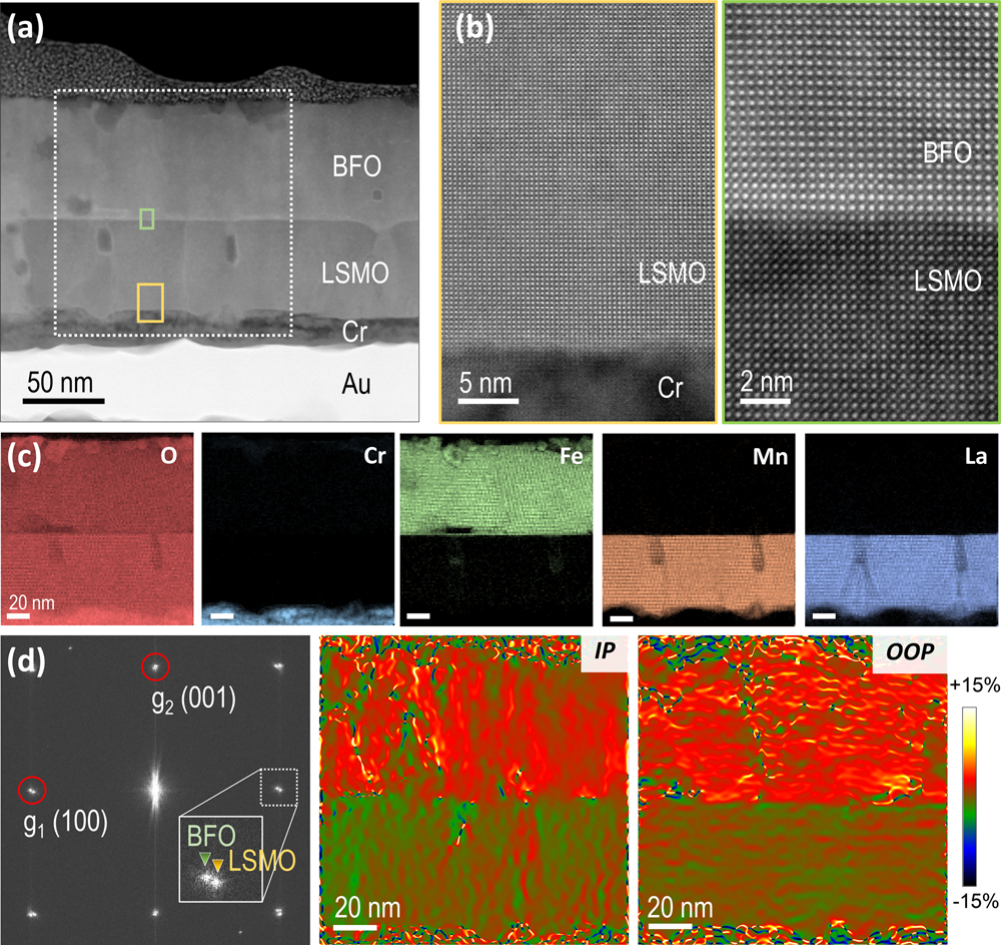
(a) Low-magnification HAADF-STEM image showing the general architecture of the BFO/LSMO membrane deposited on metal-coated-SiO_2_/Si; (b) high-resolution HAADF-STEM images of the LSMO/Cr/Au and BFO/LSMO interfaces, taken from the yellow and green squared areas in (a), respectively; (c) O, Cr, Fe, Mn, and La EELS elemental maps corresponding to the white dashed area in (a); (d) geometrical phase analysis corresponding to the white dashed area in (a), showing the strain state of the BFO layer relative to the LSMO layer (reference lattice). Left panel: FFT from (a), where the inset is a close-up image showing the splitting of the BFO and LSMO diffraction spots, indicating film relaxation. The two red circles indicate the two *g* vectors employed to compute the strain maps, *g*_1_(100) and *g*_2_(001) corresponding to the in-plane (middle panel) and out-of-plane (right panel) deformation, respectively.

The BFO/LSMO membranes were subsequently transferred to a new host rigid substrate, Cr/Au-coated SiO_2_/Si (see Figure S3 and Experimental) to investigate the microstructure and the quality of the interfaces by means of cross-sectional STEM. Figure 5a is a low-magnification HAADF image of the BFO/LSMO//Cr/Au area. Close-up atomic resolution images in Figure 5b taken at the BFO/LSMO and LSMO//Cr/Au interfaces, yellow and green boxes in Figure 5a, confirm the high epitaxial quality between the LSMO and the BFO heterostructure after the transfer process. EELS elemental mapping sustains no cation interdiffusion between the BFO and LSMO layers, Figure 5c.

To evaluate the relative strain state of the bilayers, we used geometrical phase analysis (GPA) from atomic resolution images taken at the white dashed-squared area marked in Figure 5a. Figure 5d shows the fast Fourier transformation (FFT) of the HAADF image (left panel) and the in-plane (IP) and out-of-plane (OOP) strain deformation maps (middle and right panels, respectively). As observed in the inset of the FFT, the splitting of the BFO and LSMO (100)/(001) diffraction spots indicates that the BFO is relaxed with a larger *ab* and *c* lattice parameter with respect to the LSMO, which is represented in the strain maps by the change in color in the BFO film relative to the reference LSMO lattice. In addition, from the strain maps in Figure 5d, it is observed that the BFO film exhibits inhomogeneous lattice deformations likely due to the numerous nucleation of structural defects that locally relax the BFO lattice. In fact, CSD tends to promote the formation of structural defects within the epitaxial thin film, as previously reported in other complex oxides.^39,43,44^ A more detailed analysis of these local deformations in the BFO layer by means of atomically resolved STEM images is presented in Figure S4.

**Figure 6:**
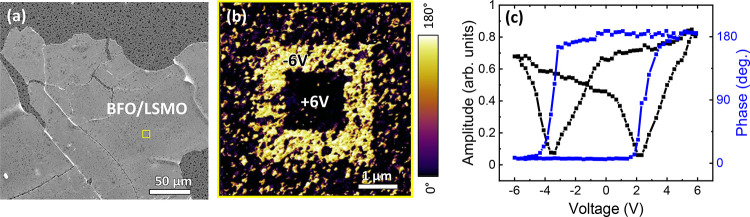
(a) SEM image of a BFO/LSMO membrane on the metal-coated SiO_2_/Si substrate; (b) PFM-phase image of a BFO/LSMO membrane; (c) amplitude and phase PFM loops.

Finally, the BFO/LSMO membranes on the same rigid substrate (Cr/Au-coated SiO_2_/Si) were probed by PFM. First note from Figure 6a that the membrane shows large continuous areas with some cracks and almost no wrinkles due to the strain release.^36,45^ The preparation of large area and morphological defect-free membranes is a challenging step where more accurate control of parameters such as strain, adhesion layer, and metal stressors could be helpful to improve the quality of the membranes.^14,45,46^ From the PFM analysis, we identified that after poling the sample at −6 and +6 V, the phase image showed a sharp contrast revealing ferroelectric behavior with retention properties, Figure 6b. We also spotted that the phase image shows noise overlapped to the 180° phase contrast due to the intermittent contact between the tip and the surface resulting from the surface topography. The corresponding PFM topographic and amplitude images recorded simultaneously with the phase images are presented in Figure S5. They demonstrate no surface degradation and suggest that the electrochemical process at the tip–surface junction during electric writing did not dominate the results. The acquired amplitude and phase signal loops showed butterfly and 180° hysteresis loops, respectively, confirming the ferroelectric nature of the material, Figure 6c.^47−49^ Note that the piezoelectric response is not quantified because the signal is recorded at near resonance. In more elaborate architectures such as SrRuO_3_/BiFeO_3_/SrRuO_3_ in which BFO presented a clear domain pattern, they observed a more dramatic change in the piezoresponse due to significant changes in the elastic constraints.^50^ To strengthen the BFO ferroelectric characterization, the PFM analysis of BFO/LSMO membranes was compared to the PFM data acquired on CSD-BFO prior to the membrane release from the PLD-LSMO/SC_2_AO//STO, under the same measurement conditions, Figure S6. A clear 180° phase contrast is also observed with a more stable signal due to better tip contact as a result of the smoother surface. When comparing the amplitude loops before and after membrane release, the amplitude obtained in the membrane is slightly larger probably related to the absence of clamping effect although small variations on the sample-tip contact from measurement to measurement make the quantification challenging. These BFO/LSMO membranes, when transferred on PET substrates (Figure 4), could be an attractive system to be further investigated under large bending strains for nanoelectromechanical systems.^27^

### Conclusions

This study unfolds the challenges of preparing (00l)-oriented BFO membranes by solution processing. The softness of the SAO sacrificial layer determines the quality of the subsequent oxide grown on top, which is critical when the oxide is deposited by solution processing. Direct solution deposition of BFO on SAO sacrificial surface produces a nanocomposite of an epitaxial BFO matrix with embedded Sr–Al–O nanoparticles. While the addition of a PLD-LSMO buffer layer prevents the formation of the nanocomposite, cation migration to the sacrificial layer occurs, hindering the release of the membrane from the substrate. Instead, the use of SrCa_2_Al_2_O_6_ offers a robust architecture to obtain crystalline BFO/LSMO membranes on both flexible PET and rigid Si substrates. The BFO is macroscopically relaxed and sustains the LSMO partially strained upon substrate release. STEM and local strain analysis on the crystalline bilayer membrane identified that the strain generated from the lattice mismatch between LSMO/BFO is gradually accommodated in the BFO by structural defects. Finally, PFM analysis confirmed the ferroelectric behavior of the CSD-BFO membrane. Therefore, the use of all-solution processed technology for oxide membranes is foreseen to be a versatile and facile ex-situ approach to prepare binary and multicomponent oxide films that can be easily implemented in a cheap manner in almost any research lab. For the particular case where water-soluble sacrifical layers are used, the procedure still requires further research to find sacrificial compositions that ensure easy etching while preserving its integrity when a precursor solution is deposited on top. The formulation of the precursor solution to identify the optimal solvent blend that is compatible with SAO is also another rich area to be explored. On the other hand, the use of solvent-free chemical deposition techniques such as atomic layer deposition could be an attractive route toward all-chemical complex oxide membranes. Finally, further investigations on the interplay between strain gradient, phase distortion, and superflexibility in these complex oxides could offer many practical applications in sensors, memories, spintronics, electronic skins, and self-powered devices.^12−14,51^ These broad range of possibilities could also be extended to other piezoelectric, ferroelectric, and multiferroic perovskite oxides anticipating a rich playground for complex oxide freestanding membranes to create new functional materials.

## Experimental Section

### Synthesis of Sr_3–*x*_Ca_*x*_Al_2_O_6_ by CSD

The preparation of Sr_3–*x*_Ca_*x*_Al_2_O_6_ films was carried out by weighing stoichiometric amounts of strontium nitrate, Sr(NO_3_)_2_ (>99%, Merck), calcium nitrate tetrahydrate, Ca(NO_3_)_2_·4H_2_O (>99%, Merck), and aluminum nitrate nonahydrate, Al(NO_3_)_3_·9H_2_O (>98%, Merck). Note that due to the high hygroscopicity of some of the precursor salts, they were stored in a glovebox as received (<0.1 ppm of O_2_ and <0.1 ppm of H_2_O). Then, the nitrate precursors were dissolved in Milli-Q water with citric acid (CA) (>99%) in a molar ratio to total metal cations (M) of 2CA:1M. The precursor solution was stirred overnight at 90 °C in a reflux condenser to obtain 0.1 M solutions. Subsequently, the solution was filtered with a poly(tetrafluoroethylene) hydrophilic filter of 0.45 μm pore size. Filtered solution was spun coated on 5 × 5 mm^2^ bare (001) SrTiO_3_ single-crystal substrates cleaned for 10 min at UV-ozone. Finally, the films were treated in a tubular furnace at 800 °C in a constant oxygen flow as described elsewhere.^34,36^ Surface film stoichiometry was verified by X-ray photoelectron spectroscopy analysis.

### Synthesis of BFO by CSD

BFO films were prepared by mixing stoichiometric amounts of bismuth nitrate, Bi(NO_3_)_3_·5H_2_O (ACS reagent >98.0%, Merck) and iron nitrate nonahydrate, Fe(NO_3_)_3_·9H_2_O (ACS reagent >98.0%, Merck) in a solvent blend 3:1 of 2-methoxyethanol and acetic acid to obtain a 0.25 M solution. Note that the metal nitrates are stored in a glovebox prior to weighing. The samples were finally annealed at 650 °C with a continuous O_2_ flow for 45 min.^30^ Multideposition was carried out to achieve 75 nm films.

### Synthesis of LSMO by PLD

To ex-situ deposit LSMO on Sr_3–*x*_Ca_*x*_Al_2_O_6_ films, the Sr_3–*x*_Ca_*x*_Al_2_O_6_ was exposed to an in-vacuum pretreatment at 825 °C for 30 min at an oxygen partial pressure, PO_2_, of 0.1 mbar. Subsequently, 60 nm of LSMO films were grown at 725 °C and a PO_2_ of 0.1 mbar using a KrF excimer laser.

### Membrane Transfer

In order to proceed with the fabrication of the membranes, a PET sheet was attached to the heterostructure, and then it was immersed in Milli Q-water. Etching time for the multilayer architecture was 1.5–2 weeks (heating at 80 °C the Milli Q-water and scratching the edge of the samples can speed up the etching time). Second transfer to Cr/Au-coated SiO_2_/Si and conductive substrates required to apply 70–100 °C for 10–30 min. Then, the transferred architectures were exposed to 500–600 °C for 1 h in 0.6 L·min^–1^ O_2_ flow to improve the contact surface. The entire transfer process is schematized in Figure S3.

### Crystal Structure

XRD measurements were performed with Cu–Kα using a Bruker-AXS instrument (model A25 D8 Discover). Cross-sectional specimens for STEM investigation were prepared using the standard focused ion beam (FIB) lift-out process in a Thermo Fisher Scientific FIB system. Protective Pt layers were applied over the region of interest before cutting and milling. To minimize the sidewall damage and ensure a sufficiently thin specimen for electron transparency, final milling was carried out at a voltage of 2 kV. Aberration-corrected STEM imaging was performed using a Nion HERMES-100, operated at 60 kV, at the University of Chinese Academy of Sciences, Beijing, China. HAADF images were acquired using an annular detector with a collection semiangle of 75–210 mrad. To minimize the possible beam-induced structural damage on the Sr_3–*x*_Ca_*x*_Al_2_O_6_ sacrificial films, images were acquired with a reduced beam current (5 pA). EELS measurements were performed using a collection semiangle of 75 mrad, an energy dispersion of 0.9 eV per channel, and a probe current of ∼20 pA. To analyze quantitatively the displacement of both Fe atoms as a function of the strain gradient, we precisely measured their atomic positions from the HAADF images using Atomap,^52^ a Python library for analyzing atomic resolution images relying on fitting 2-D Gaussian functions to every atomic column in an image and finding all major symmetry axes.

### PFM Characterization

Piezoelectric force microscopy (PFM) measurements were performed with an MFP-3D microscope (Oxford Instrument Co.), using the BudgetSensors silicon (n-type) probe with a Pt coating (Multi75E-G). Scanned areas were 5 × 5 μm^2^ and the electrically written regions were 3 × 3 μm^2^. To achieve better sensitivity, the dual-frequency resonance-tracking (DART) mode was employed.^53,54^ PFM voltage hysteresis loops were always performed at remanence using a dwell time of 100 ms. The quantification of the piezo coefficient using DART is difficult due to the simultaneous variation of measurement frequency and the variation of the maxima of the resonance amplitude while measuring; consequently, arbitrary units (a.u.) are indicated in the amplitude of the piezoresponse.

## Supporting Information Available

XRD structural analysis of BFO/SAO//STO and the corresponding AFM topography; XRD analysis of BFO/SC_2_AO//STO; sketch of the process to prepare bilayer membranes; texture analysis of the BFO and LSMO films in the heterostructure and membrane; strain analysis of the BFO/LSMO bilayer membrane and local displacements of the atoms; and PFM analysis of the BFO layer before and after membrane release (PDF)
