# Supplementary material for: Unfolding the Challenges To Prepare Single Crystalline Complex Oxide Membranes by Solution Processing
Source: ACS Appl Mater Interfaces. 2024 Jul 5;16(28):36796–803. doi: 10.1021/acsami.4c05013 (PMC11261560; doi:10.1021/acsami.4c05013)
Supplement: Supplementary file 1 — am4c05013_si_001.pdf [file am4c05013_si_001.pdf]

Supporting Information for Unfolding the challenges to prepare single crystalline complex oxide membranes by solution processing

Pol Salles,[†,¶] Roger Guzmán,[‡,¶] Huan Tan,[†] Martí Ramis,[†] Ignasi Fina,[†] Pamela Machado,[†] Florencio Sánchez,[†] Gabriele De Luca,[†] Wu Zhou,[‡] and Mariona Coll[*,†]

†*ICMAB-CSIC, Campus UAB 08193, Bellaterra, Barcelona, Spain*

‡*School of Physical Sciences, University of Chinese Academy of Sciences, Beijing 100049, China*

¶*Contributed equally to this work*

E-mail: mcoll@icmab.es

Phone: +34 93 5801853

CSD-BFO/CSD-SAO//STO

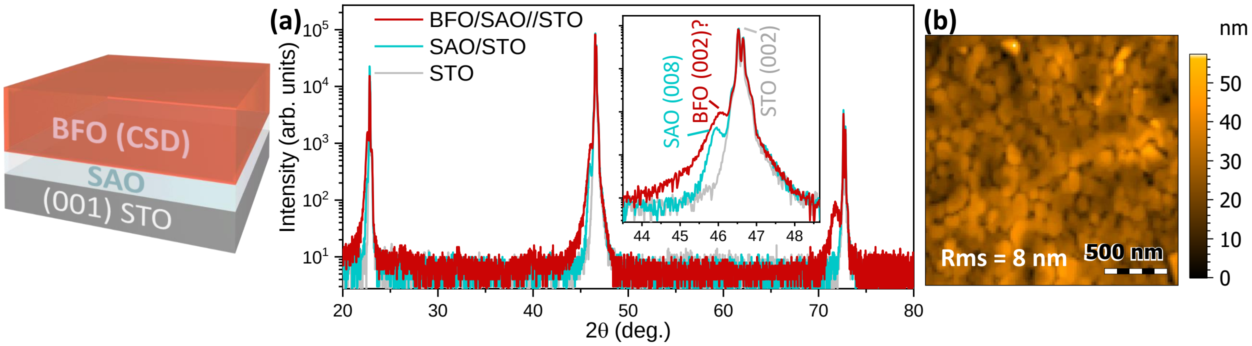

Figure S1: (a) XRD θ- 2θ scan of CSD-BFO deposited on CSD-SAO//STO; (b) corresponding AFM topographic image

CSD-BFO/CSD-SC_2AO//STO

The XRD θ- 2θ scan shown in Figure S2 compares the spectra of the SC_2AO//STO and BFO/SC_2AO//STO samples. The (008) Bragg reflection of SC_2AO observed on STO substrate disappears after the deposition of BFO while a peak at 45.81º assigned to BFO is identified.

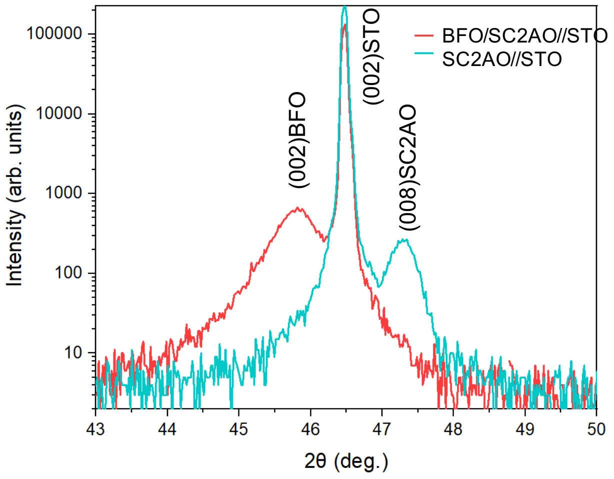

Figure S2: XRD θ-2θ scan of BFO-CSD/SC_2AO on STO and compared to SC_2AO on STO

Scheme of the process to prepare bilayer membranes

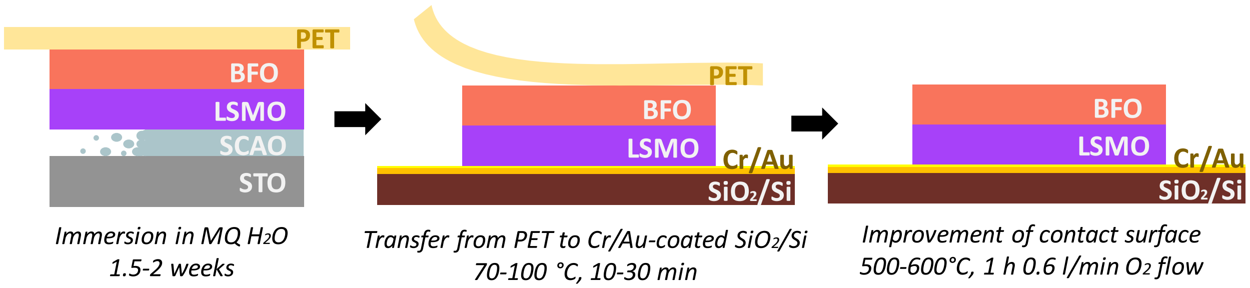

Figure S3: Scheme of the process followed to transfer the BFO/LSMO membrane from its initial BFO/LSMO/SC_2AO//STO heterostructure to the Cr/Au-coated Si wafer.

Table S1: Texture analysis of BFO and LSMO films before and after membrane release from the rocking curve ($\Delta\omega$). In parenthesis it is indicated the Bragg reflection from which the full width at half maximum of the ω-scan has been calculated. In bold it is indicated the measured component. Note that the (002) Bragg reflection from LSMO when grown on SC_2AO appears as a shoulder in the STO substrate and hampers the rocking curve analysis.

	$\Delta\omega$ ±0.02	**Heterostructure**	$\Delta\omega$ ±0.02	**Membrane on PET**	$\Delta\omega$ ±0.02
**STO**	0.02 (002)	-	-	-	
**SC_2AO**//STO	0.15 (008)	LSMO/**SC_2AO**//STO	0.19 (008)	-	
**LSMO**//STO	0.09 (002)	**LSMO**/SC_2AO//STO	-	-	
**BFO**//STO	0.71 (002)	**BFO**/LSMO/SC_2AO//STO	1.20 (002)	**BFO**/LSMO	1.30 (002)
				BFO/**LSMO**	0.90 (002)

GPA analysis of the local deformations of BFO in the bilayer membrane

Figure S4a shows the local deformation map of an enclosed BFO area where the IP and OOP deformation maps clearly show the presence of misfit dislocations near the BFO/LSMO interface (white dashed circles) and the formation of a vertical extended defect (white dashed line) also nucleating at the interface. Here, the reference lattice (zero deformation) is set at the central part of the film, far from the influence of defects. Figure S4b shows an enlarged HAADF-STEM image from the central part of the film (dashed box in Figure S4a) where the BFO lattice undergoes a horizontal strain gradient deformation, increasing from left to right from $\sim$ 0% to up to +1.4% in-plane and -1.4% out-of-plane strain. The deformation

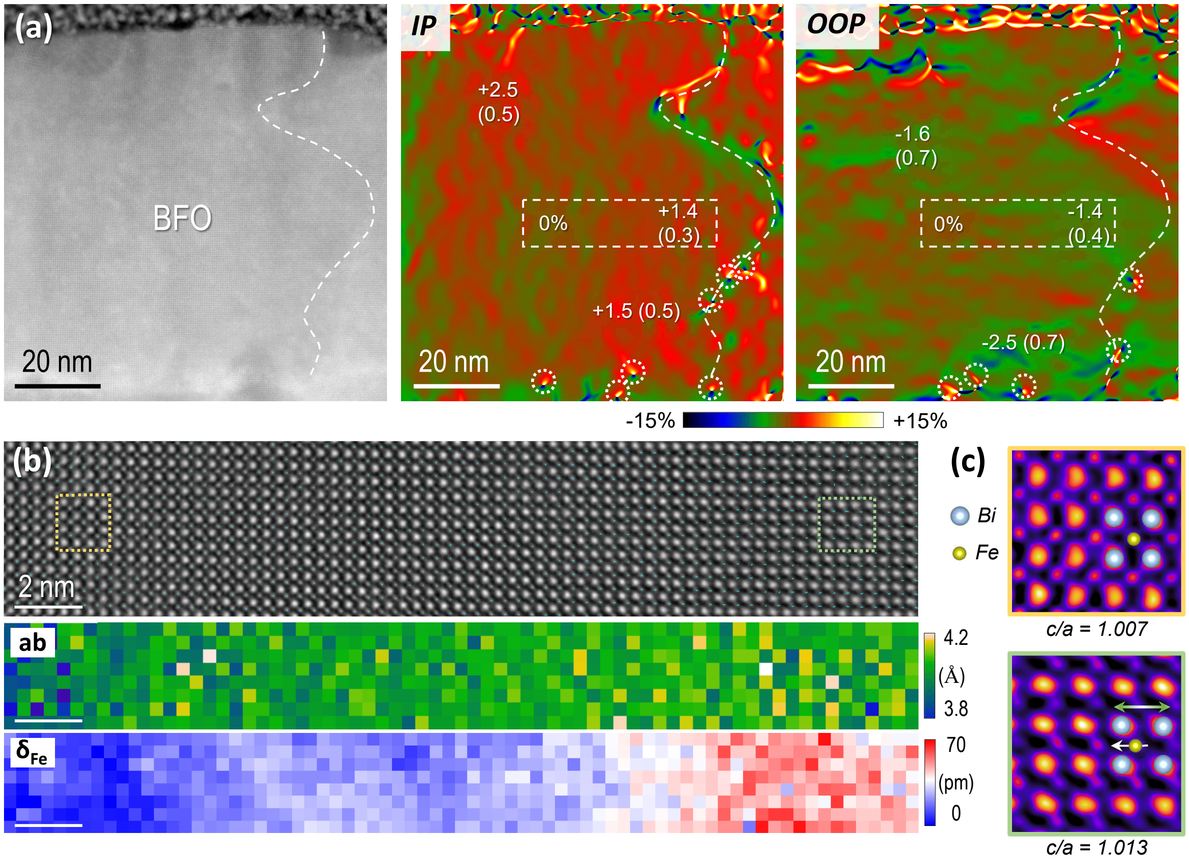

Figure S4: (a) Geometrical phase analysis of the BFO phase in the BFO/LSMO membrane, computed from the HAADF image in the left panel. The middle and right panels are the in-plane (IP) and out-of-plan (OOP) deformation maps, respectively, where the reference lattice is taken in the central part of the film, where the reference strain value is set as zero. The vertical dashed line indicates an extended defect, while the dashed circles are misfit dislocations. (b) Mapping of the Fe atomic displacements in the BFO upon a horizontal strain gradient deformation, increasing from left to right. The vector map is overlaid on the atomically resolved HAADF image (top panel) taken from the dashed area in (a). The displacement of the Fe atoms from the center of the unit cell, δ_{Fe}, are indicated with the length, direction and modulus of the vector lines. The middle panel corresponds to the lattice parameter analysis along the in-plane direction, while the bottom panel is the displacement map, δ_{Fe}, of the B-site (Fe) atoms relative to the center of four neighboring A-site (Bi) ions. (c) Close up images taken from the dashed squares in (b) showing the direction of the Fe ions off-centering (white arrow) upon distortion (green arrow).

is mapped in the middle panel, showing a change in the lattice parameter from ~ 3.93 $\AA$ to ~ 4.01 $\AA$, which barely modifies the tetragonality c/a from ~ 1.007 to 1.013, from left to right, respectively. This deformation is smaller than that reported for significantly thinner unbent BFO membranes, i.e. one-two unit cells, (c/a$\sim$1.22)[1] or for BFO films under large

compressive strain (c/a∼1.25).[2,3] Therefore, differences in membrane thickness and the use of a solution processing promotes the formation of relaxed membranes.

PFM analysis

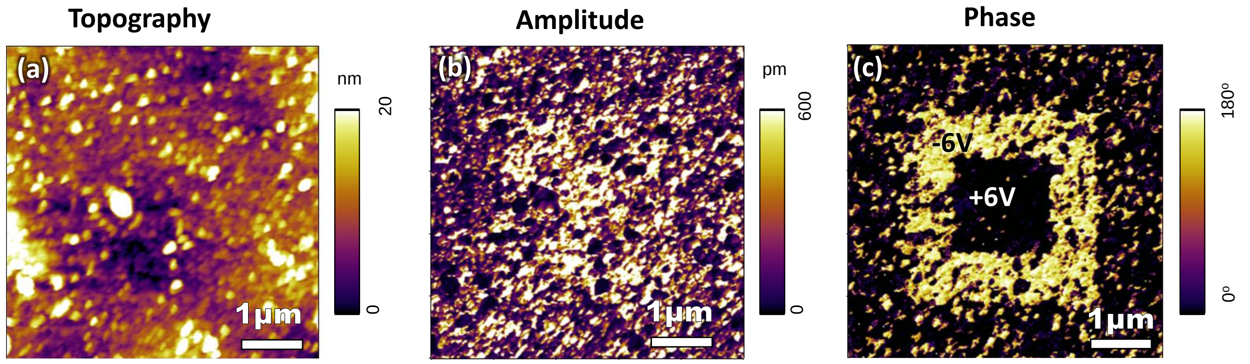

Figure S5: (a) AFM topography, (b) PFM amplitude and (c) PFM phase images obtained simultaneously from a BFO/LSMO membrane released from SC_2AO and stamped on $Au/SiO_2/Si$.

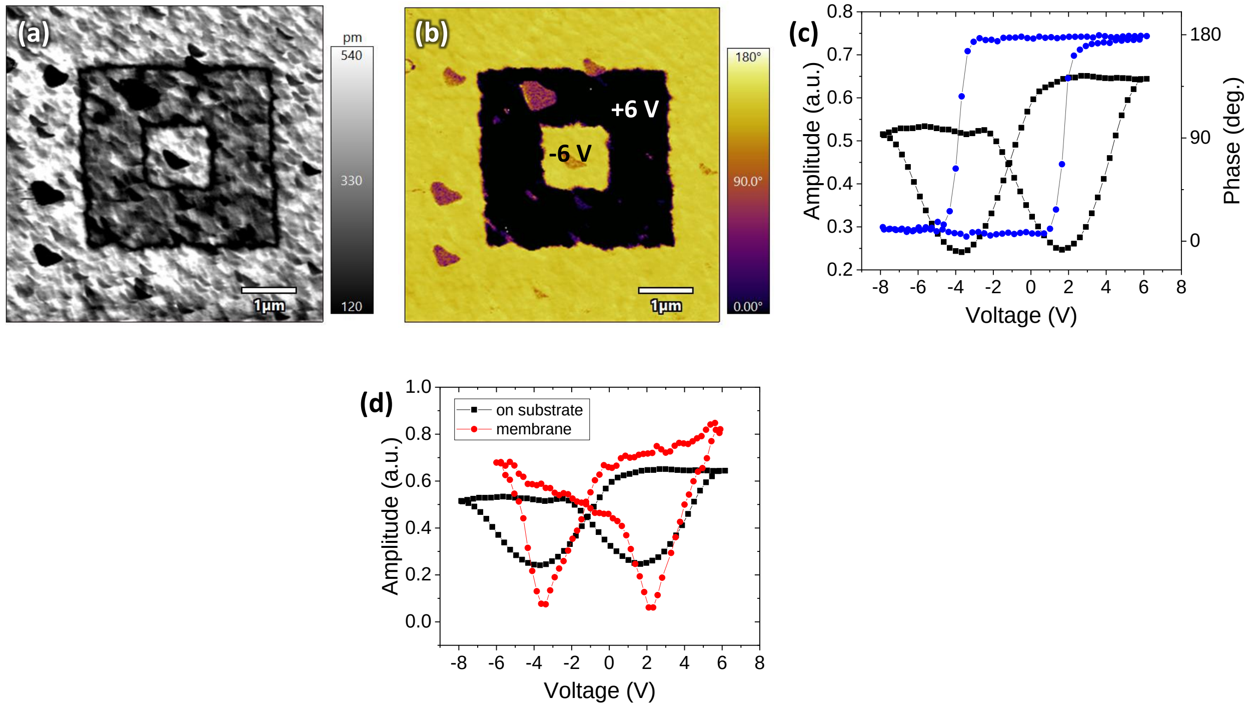

Figure S6: (a) PFM amplitude, (b) PFM phase image and (c) Phase and amplitude loops of the $BFO/LSMO/SC_2AO//STO$; (d) Amplitude loop comparison between a released BFO/LSMO membrane and the BFO/LSMO still clamped on $SC_2AO//STO$

Figure S6 shows the PFM analysis from the CSD-BFO film on PLD-LSMO/SC_2AO//STO prior membrane release. From the phase image (Figure S6b) it is clearly identified a 180º phase contrast when applying +6V and -6V in the squared areas. The amplitude and phase loops from Figure S6c confirm the ferroelectric behavior. From the amplitude and phase images it is identified triangular shapes that can be attributed to the presence of iron oxide particles. Figure S6d shows comparison of piezoelectric amplitude loop in a film on SC_2AO and in a membrane measured in similar conditions. It can be observed that the signal is more stable before the release due to better tip contact as a result of the smoother surface. The amplitude in the membrane is slightly larger probably related to the absence of the clamping effect. However, small variations on the sample-tip contact from measurement to measurement make the quantification difficult and can also contribute to the small observed variation.

References

(1) Ji, D.; Cai, S.; Paudel, T. R.; Sun, H.; Zhang, C.; Han, L.; Wei, Y.; Zang, Y.; Gu, M.; Zhang, Y.; Gao, W.; Huyan, H.; Guo, W.; Wu, D.; Gu, Z.; Tsymbal, E. Y.; Wang, P.; Nie, Y.; Pan, X. Freestanding crystalline oxide perovskites down to the monolayer limit. *Nature* **2019**, *570*, 87–90.

(2) Zhang, J. X.; He, Q.; Trassin, M.; Luo, W.; Yi, D.; Rossell, M. D.; Yu, P.; You, L.; Wang, C. H.; Kuo, C. Y.; Heron, J. T.; Hu, Z.; Zeches, R. J.; Lin, H. J.; Tanaka, A.; Chen, C. T.; Tjeng, L. H.; Chu, Y.-H.; Ramesh, R. Microscopic origin of the giant ferroelectric polarization in tetragonal-like $BiFeO_3$. *Phy. Rev. Lett.* **2011**, *107*, 147602.

(3) Béa, H.; Dupé, B.; Fusil, S.; Mattana, R.; Jacquet, E.; Warot-Fonrose, B.; Wilhelm, F.; Rogalev, A.; Petit, S.; Cros, V.; Anane, A.; Petroff, F.; Bouzehouane, K.; Geneste, G.; Dkhil, B.; Lisenkov, S.; Ponomareva, I.; Bellaiche, L.; Bibes, M.; Barthélémy, A. Evidence for Room-Temperature Multiferroicity in a Compound with a Giant Axial Ratio. *Phys. Rev.Lett.* **2009**, *102*, 217603.
